# Interocular symmetry, intraobserver repeatability, and interobserver reliability of cone density measurements in the 13-lined ground squirrel

**DOI:** 10.1371/journal.pone.0223110

**Published:** 2019-09-26

**Authors:** Benjamin S. Sajdak, Alexander E. Salmon, Rachel E. Linderman, Jenna A. Cava, Heather Heitkotter, Joseph Carroll

**Affiliations:** 1 Ophthalmology & Visual Sciences, Medical College of Wisconsin, Milwaukee, WI, United States of America; 2 Cell Biology, Neurobiology and Anatomy, Medical College of Wisconsin, Milwaukee, WI, United States of America; 3 Morgridge Institute of Research, Madison, WI, United States of America; 4 Biophysics, Medical College of Wisconsin, Milwaukee, WI, United States of America; National Eye Centre, UNITED STATES

## Abstract

**Background:**

The 13-lined ground squirrel (13-LGS) possesses a cone-dominant retina that is highly amenable to non-invasive high-resolution retinal imaging. The ability for longitudinal assessment of a cone-dominant photoreceptor mosaic with an adaptive optics scanning light ophthalmoscope (AOSLO) has positioned the 13-LGS to become an accessible model for vision research. Here, we examine the interocular symmetry, repeatability, and reliability of cone density measurements in the 13-LGS.

**Methods:**

Thirteen 13-LGS (18 eyes) were imaged along the vertical meridian with a custom AOSLO. Regions of interest were selected superior and inferior to the optic nerve head, including the cone-rich visual streak. Non-confocal split-detection was used to capture images of the cone mosaic. Five masked observers each manually identified photoreceptors for 26 images three times and corrected an algorithm’s cell identification outputs for all 214 images three times. Intraobserver repeatability and interobserver reliability of cone density were characterized using data collected from all five observers, while interocular symmetry was assessed in five animals using the average values of all observers. The distribution of image quality for all images in this study was assessed with open-sourced software.

**Results:**

Manual identification was less repeatable than semi-automated correction for four of the five observers. Excellent repeatability was seen from all observers (ICC = 0.997–0.999), and there was good agreement between repeat cell identification corrections in all five observers (range: 9.43–25.71 cells/degree^2^). Reliability of cell identification was significantly different in two of the five observers, and worst in images taken from hibernating 13-LGS. Interocular symmetry of cone density was seen in the five 13-LGS assessed. Image quality was variable between blur- and pixel intensity-based metrics.

**Conclusions:**

Interocular symmetry with repeatable cone density measurements suggest that the 13-LGS is well-suited for longitudinal examination of the cone mosaic using split-detection AOSLO. Differences in reliability highlight the importance of observer training and automation of AOSLO cell detection. Cone density measurements from hibernating 13-LGS are not repeatable. Additional studies are warranted to assess other metrics of cone health to detect deviations from normal 13-LGS in future models of cone disorder in this species.

## Introduction

Development and translation of treatment strategies for diseases involving cone degeneration has been limited by accessibility of animal models that mimic human pathophysiology. There are several cone photoreceptor disorder models involving nocturnal mice and rats [1], despite these animals having sparse cone mosaics. As they possess a cone-exclusive fovea, non-human primates like the macaque and marmoset may be more appropriate models [2], but the cost and logistics of maintaining non-human primates is too burdensome for most research institutions. We have been examining a supplemental strategy of studying small, diurnal, cone-dominant mammalian models for investigation of retinal health and disease. For example, the 13-lined ground squirrel (13-LGS) is a cone-dominant mammal (~85% cones) that is highly amenable to non-invasive imaging of the cone mosaic using adaptive optics scanning light ophthalmoscopy (AOSLO) [3, 4]. Central to determining the suitability of these animals as models for studying cone structure in health and disease is assessing the repeatability and reliability of quantitative measurements of the cone mosaic.

The repeatability and reliability of AOSLO cone density measurements has been described in visually normal subjects [5–7] and patients with inherited retinal diseases [8–11]. Cone density measurement validation has been lacking in studies using animal models, with the exception of validating OCT-derived cone density measurements in zebrafish [12]. Density is a common metric for assessing the cone mosaic and will be critical for longitudinal assessment and interpretation of pathology in animal models of cone disorder. Reliable identification of cones is vital for accurate density measurements, which can be challenging with confocal AOSLO due to varying reflectance intensity of cones [13–15]. The advent of non-confocal split-detection has enabled more reliable detection of cones in the human perifovea [16], and in the 13-LGS retina [4]. Automated algorithms to identify cones in split-detection AOSLO images have been validated in visually normal subjects [17–19], patients with achromatopsia [20], and patients with Stargardt disease [18, 21], but have yet to be validated in animal models.

The present study evaluates the intraobserver repeatability and interobserver reliability of cone density measurements from split-detection AOSLO images captured in the 13-LGS. We also evaluated the interocular symmetry of cone density using the average of all observer measurements. These data will be useful for evaluating future 13-LGS models and the effect of subsequent experimental treatments.

## Methods

### Animals

Thirteen 13-LGS (*Ictidomys tridecemlineatus*; 9 female, 4 male) were obtained from the University of Wisconsin-Oshkosh Squirrel Colony for use in this study at the Medical College of Wisconsin. Animal husbandry and dietary protocols were provided according to the Oshkosh Squirrel Colony guidelines [22]. The experimental procedures described were approved by the Institutional Animal Care and Use Committee of the Medical College of Wisconsin and were in accordance with the ARVO Statement for the Use of Animals in Ophthalmic and Vision Research. None of the animals were sacrificed upon completion of this study.

#### Seasonal set

Five 13-LGS were used for longitudinal assessment of the cone mosaic throughout the hibernation cycle from a previous study [23]. These 13-LGS were included to encompass the range of image quality that results from natural seasonal variability. Imaging for these animals was performed longitudinally at distinct physiological states of the 13-LGS seasonal cycle (pre-hibernation, torpor, and post-hibernation euthermia), between the hours of 10 AM and 3 PM from October to March. Additional information regarding the husbandry and monitoring of these animals imaged throughout hibernation is detailed elsewhere [23]. These 5 squirrels were 5–10 months old.

#### Euthermic set

Eight 13-LGS were in a euthermic state when imaged to avoid potential seasonal confounds from this species’ annual hibernation cycle, which has an effect on non-invasive retinal imaging procedures [23, 24]. Imaging for these animals was performed once, between the hours of 10 AM and 3 PM from June to September. At the time of retinal imaging 3 squirrels were 4 months old, 2 squirrels were 15 months old, and 3 squirrels were 27 months old.

### Adaptive optics scanning light ophthalmoscopy (AOSLO)

The 13-LGS photoreceptor mosaic was imaged with non-confocal split-detection AOSLO [16] using a custom instrument optimized for 13-LGS imaging (4.5 mm subject pupil). Animals were imaged under inhaled isoflurane anesthesia (5% induction, 4–5% maintenance in 1L/min O_2_ flow; torpid animals did not require induction but were maintained on 2% isoflurane in 0.5L/min O_2_ flow). Pupil dilation and cycloplegia were induced with 1% tropicamide and 2.5% phenylephrine, and the eyelids were held open with a pediatric ocular speculum. Saline or artificial tears were used to maintain corneal hydration throughout imaging. The imaging protocol started at the optic nerve head (ONH): the horizontal ONH stretches across ~8mm of the posterior pole of the 13-LGS eye and serves as a landmark that divides superior from inferior retina. Image sequences were captured up to 10 degrees superiorly and 20 degrees inferiorly relative to the ONH. The scale of the AOSLO images was calculated using Ronchi gratings to determine the pixels per degree for the field of view captured during each imaging session.

### Analyzing the 13-LGS photoreceptor mosaic

**Fig 1:**
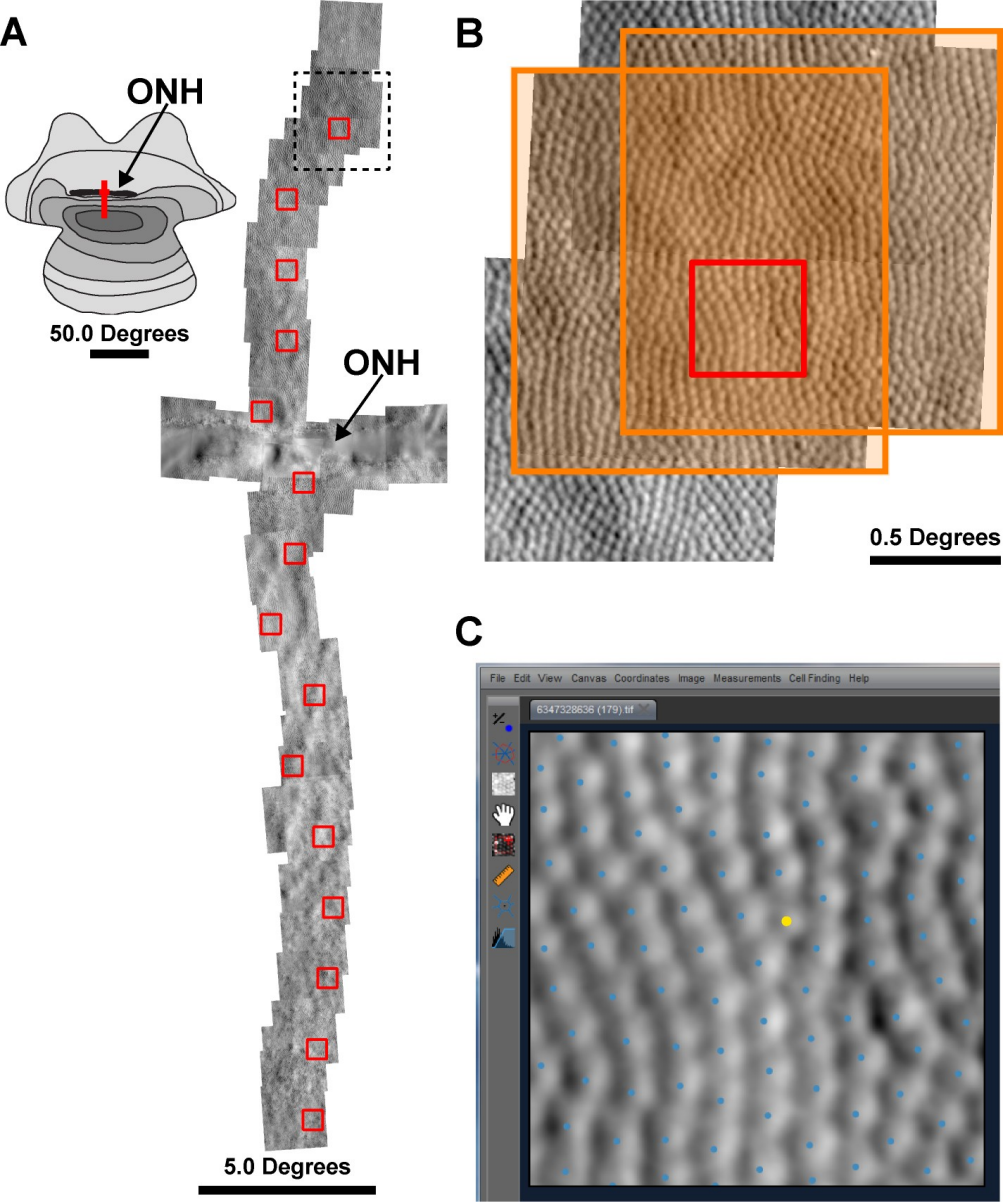
Region of Interest (ROI) and cell identification. (A) Schematic (photoreceptor density map reproduced from Long & Fisher [1983]) showing approximate retinal location (red line) of split-detection AOSLO montage relative to the optic nerve head (ONH). For each montage, ROIs (red boxes) were selected at 2-degree intervals. (B) A zoomed-in section from the montage in (A), outlined by the dashed line. The orange boxes indicate two available images that contain the entire ROI. Once the ROI is cropped from one of the images, the cone segmentation algorithm is applied to the ROI. (C) Example of an ROI (from panel [B]) with cone segmentation results in the observer interface for photoreceptor identification correction. In some cases, rods may be distinguished based on their smaller size relative to cones (rod marked as a yellow dot); rods were included in the analysis.

Reference frames were automatically selected from image sequences [25], then 80–150 frames were registered and averaged (**S1 Video** ([26]). The resulting images were automatically montaged (github.com/BrainardLab/AOAutomontaging; Version 1.5; [27]), with manual correction to the resulting alignment performed in Photoshop CS6 (Adobe, San Jose, CA). Regions of interest (ROIs) were extracted from the montages at 2-degree intervals up to 10 degrees superior and 20 degrees inferior from the ONH using custom software (Translational Imaging Innovations, Inc., Hickory, NC; **Fig 1A**). The ROIs from the seasonal set we taken from the same 2-degree superior location longitudinally. Each 0.55 x 0.55° ROI was cropped from a single image at each retinal location (**Fig 1B**), and the ROI selector was moved away from blood vessels that obscure the photoreceptor mosaic. The seasonal image set consisted of 26 images (five animals, left eye, 5–6 images per animal), and the euthermic image set consisted of 188 images (eight animals, one or both eyes, 13–30 images per animal). Five observers with varying levels of expertise in analyzing AOSLO photoreceptor mosaics were selected to review and identify 13-LGS photoreceptors. Each observer was introduced to the tools of the cone counting program, which includes a brightness and contrast histogram adjustment, a “flag missing cells” algorithm, and a Voronoi diagram overlay. The 26 images from the seasonal set were presented in a random order and the observer was masked from any information regarding animal, physiological state, and retinal location. Photoreceptors were then manually identified in these 26 images by each observer three times using the cone counting program interface (**Fig 1C**). Photoreceptors were automatically detected in all 214 images using an adaptive filtering and local detection algorithm [17]. After automated detection of 13-LGS photoreceptors, the five observers were presented the masked images in random order and identified photoreceptors missed by the program. All 214 images were corrected by each observer three times; once the set of images was corrected to the best of the observer’s ability, they moved on to the next copy of the set. No time restrictions were given for this experiment. Infrequent rod photoreceptors have slightly smaller inner segment diameters compared to cones in the 13-LGS [28], but cannot dependably be distinguished from cones in the central retina (**Fig 1C**), so they were unavoidably included in the analysis. Density was calculated by dividing the number of bound Voronoi cells by their summed area [29].

### Image quality analysis

All 214 images were imported into the CellProfiler software [30] and analyzed using the ‘Measure Image Quality’ module [31]. ‘Focus Score’ measures pixel intensity variance across the image using a normalized variance algorithm [30, 32, 33]. ‘Power Log-Log Slope’ measures the slope of the image log-log power spectrum. ‘Std Intensity’ measures the standard deviation of pixel intensity values. ‘MAD Intensity’ measures the median absolute deviation of pixel intensity values.

### Statistical methods

Fully manual segmentations were performed on the 26 images in the seasonal set three times by each of the five observers to compare to algorithm performance on 13-LGS images (26 x 3 x 5 = 390). The total image set (n = 214 images) was corrected after algorithm segmentation three times by each of the five observers, resulting in 3,210 observations for analysis (214 x 3 x 5 = 3,210). Within-observer standard deviation (*S*_*w*_) was used to calculate the repeatability coefficient (2.77•*S*_*w*_) and measurement error (1.96•*S*_*w*_) [34]. Repeatability is reported both with the cone density measurements in cells/degree^2^ and as a percentage of the mean cone density for each observer. Photoreceptor density was compared between sexes and ages from the euthermic image set using a Sidak’s multiple comparisons test. Interocular symmetry bias, limits of agreement, and 95% confidence intervals were calculated using methods described by Bland and Altman [35–37]. Image quality metric correlations to variance in density measures were tested with Spearman’s R Test. Data were tested for normality using the Shapiro-Wilk test. Calculations were completed using Microsoft Excel (2016, Version 1803) and Prism version 8.1.1 (GraphPad, La Jolla, CA).

## Results

### Fully manual photoreceptor identification compared to semi-automated correction

To evaluate the performance of the segmentation algorithm compared to manual cell selection, we compared the repeatability of fully manual observer photoreceptor identification to the segmentation algorithm output using the seasonal set of 26 images (**Table 1**). The fully manual photoreceptor identifications were less repeatable overall, with repeatability coefficients ranging from 25.92 to 75.08 cells/degree^2^ (or 8.09–26.14%), compared to the repeatability coefficients ranging from 18.01 to 59.09 cells/degree^2^ (or 4.21–15.72%) after semi-automated photoreceptor identification correction. Observer performance varied between the photoreceptor identification techniques, with only observer 4 having better repeatability in the manual photoreceptor identification task.

**Table 1:** Repeatability of 13-LGS photoreceptor density measurements in fully manual photoreceptor identification compared to semi-automated correction.

		Observer 1	Observer 2	Observer 3	Observer 4	Observer 5
Manual	Repeatability Coefficient^#^^,^* (95% CI)	25.92 (24.06–27.77)	61.99 (57.56–66.42)	75.08 (69.72–80.45)	45.36 (42.11–48.60)	39.03 (36.24–41.82)
	Measurement Error^^^^,^*	18.34	43.86	53.13	32.09	27.61
Semi-Auto	Repeatability Coefficient^#^^,^* (95% CI)	15.58 (14.46–16.69)	18.01 (16.72–19.29)	28.03 (26.03–30.03)	59.09 (54.87–63.31)	15.94 (14.80–17.08)
	Measurement Error^^^^,^*	11.02	12.74	19.83	41.81	11.28

*Density values in cells/degrees^2^

^#^The difference between two measurements by that observer for the same image is expected to be less than this for 95% of paired observations.

^^^The difference between a measurement and the true value is expected to be less than this for 95% of observations.

### Repeatability and reliability of 13-LGS photoreceptor density measurements

Intraobserver repeatability of photoreceptor density measurements was excellent for all observers in this study (ICC = 0.997–0.999, **Table 2**), suggesting that 99.7% of variance is due to differences in photoreceptor density across different animals, physiological states, and/or retinal locations. However, to assess the magnitude of variance of repeated measurements for this study, we analyzed within-observer standard deviation (*S*_*w*_). Based on the three readings on 214 images by each observer, the repeatability coefficients (as absolute differences) ranged from 9.43 to 25.71 cells/degree^2^ (or 1.99–5.55%), which provides an estimate of what the differences would be between two photoreceptor density measurements for 95% of occasions (**Table 2**). These data suggest that the detectable magnitude of cell loss in these animals would be around 28 cells/degree^2^ depending on the observer. While all observers’ measurements can be considered highly repeatable, observer 1 had the best repeatability, followed by observers 5, 3, 2, and 4 (**Table 2**).

**Table 2:** Repeatability and reliability of 13-LGS photoreceptor density measurements.

	Observer 1	Observer 2	Observer 3	Observer 4	Observer 5
Range of Means*	272–885	271–876	166–867	265–881	263–877
Repeatability Coefficient^#^^,^* (95% CI)	9.43 (8.76–10.11)	13.49 (12.52–14.45)	13.23 (12.28–14.17)	25.71 (23.87–27.55)	10.61 (9.85–11.37)
Measurement Error^^^^,^*	6.68	9.54	9.36	18.19	7.51
ICC	0.9996	0.9991	0.9988	0.9967	0.9994
(95% CI)	(0.9994–09997)	(0.9989–0.9993)	(0.9985–0.9990)	(0.9959–0.9974)	(0.9993–0.9995)

*Density values in cells/degrees^2^

^#^The difference between two measurements by that observer for the same image is expected to be less than this for 95% of paired observations.

^^^The difference between a measurement and the true value is expected to be less than this for 95% of observations.

We wanted to assess any density-dependent trends since the dataset contained images throughout the vertical meridian, which varies substantially in photoreceptor density (Range of means = 166–885 cells/degree^2^;**Table 2**). Despite the range of photoreceptor densities, there was not a significant trend when comparing the total mean photoreceptor density (derived from 15 observations across all observers) and standard deviation for all 214 images (p = 0.456, linear regression; **S1 Fig**), suggesting there was no density-dependent bias when analyzing the observers as a group. The standard deviation of each observer’s measurements corresponded to the repeatability values in **Table 2**, in that more images had a larger standard deviation in observers with worse repeatability (most notably with observer 4; **S1 Fig**).

Interobserver reliability was assessed by comparing the average values for each image from each of the 5 observers. These values did not pass normality testing for any observer (p < 0.0001, Shapiro-Wilk), so the Friedman test was used as a nonparametric alternative to a one-way ANOVA. The observers were significantly different from one another (p < 0.0001, Friedman test), and multiple comparison post testing revealed any pair involving observers 3 or 5 were significantly different (p < 0.0001, Dunn’s multiple comparisons test). There was no obvious difference in the training or level of experience of observers 3 and 5.

**Fig 2:**
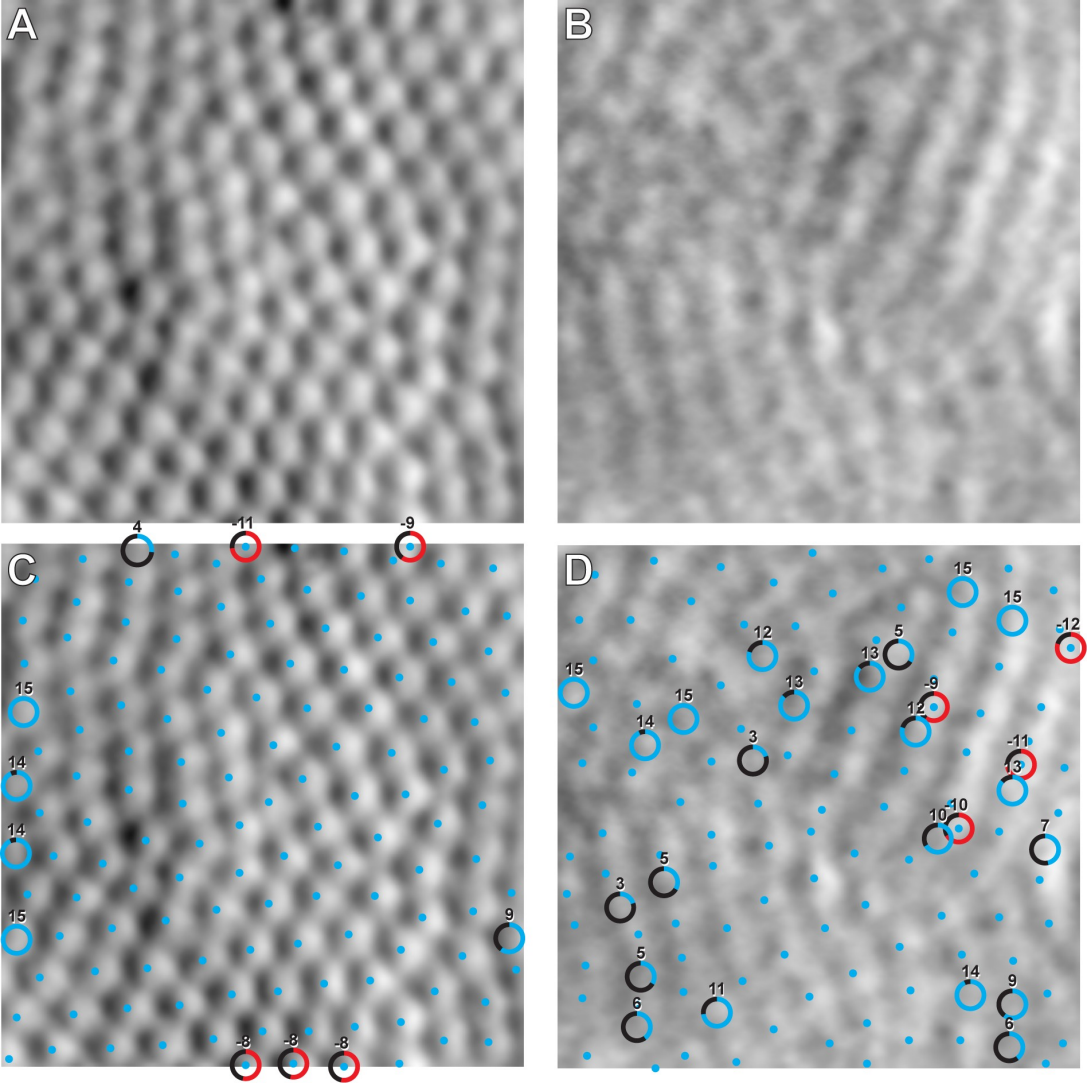
Extremes of interobserver agreement. (A) The image with the highest agreement (lowest variance) in photoreceptor identification. (B) The image with the lowest agreement (highest variance) in photoreceptor identification from the euthermic image set. (C) and (D) show the results of the cone segmentation algorithm (blue dots) with observer corrections. Circles indicate photoreceptors that were added (blue gradient) or removed (red gradient) by more than one observer, and the number outside the circle denotes how many times the photoreceptor was added or removed (15 being the maximum). In panel (D), three out of four commonly removed cells overlapped with added cells, suggesting that these were cells simply shifted to a new location rather than truly being added or removed.

For each image, we examined the addition and removal of cones by each observer following automated processing by the automated algorithm. The mean ± SD number of cells added was 5.15 ± 2.80 across all 3,210 measurements and the mean ± SD number of cells removed was 4.56 ± 3.61. On average, observers shifted the location of the cell center estimated by the algorithm for 5% - 22% of cells. For several images, the number of cells added was similar to the number of cells removed (**Fig 2A and 2C**), offsetting any major effect on cone density. In high quality images (e.g. **Fig 2A**), cells added or removed by the observer were mostly around the edges of the image, and even then, such differences are unlikely to affect the computed density as we are using bound Voronoi cells to compute density (**Fig 2C**). In low quality images (e.g. **Fig 2B**), automated cell identification was unable to find the necessary contrast features of the image and more subjective and variable observer cell addition/removal was needed (**Fig 2D**).

### Distribution of image quality

In order to objectively assess the distribution of image quality independent of cell identification, we measured image qualities through CellProfiler, which measures a suite of unique image features. **S2 Fig** shows the distribution of image quality for all 214 images for these 4 image quality metrics. Image quality was variable between blur- and pixel intensity-based metrics. There was a negative association between variance (from all 15 observations for all 214 images) and ‘Focus Score’, ‘Power Log-Log Slope’, ‘Std Intensity’, and ‘MAD Intensity’ (r_s_ = -0.319, -0.320, -0.349, and -0.3879, respectively; *p* < 0.0001; Spearman Correlation). This demonstrates that the agreement between observers was worse for images of lower image quality.

### Interocular symmetry

**Fig 3:**
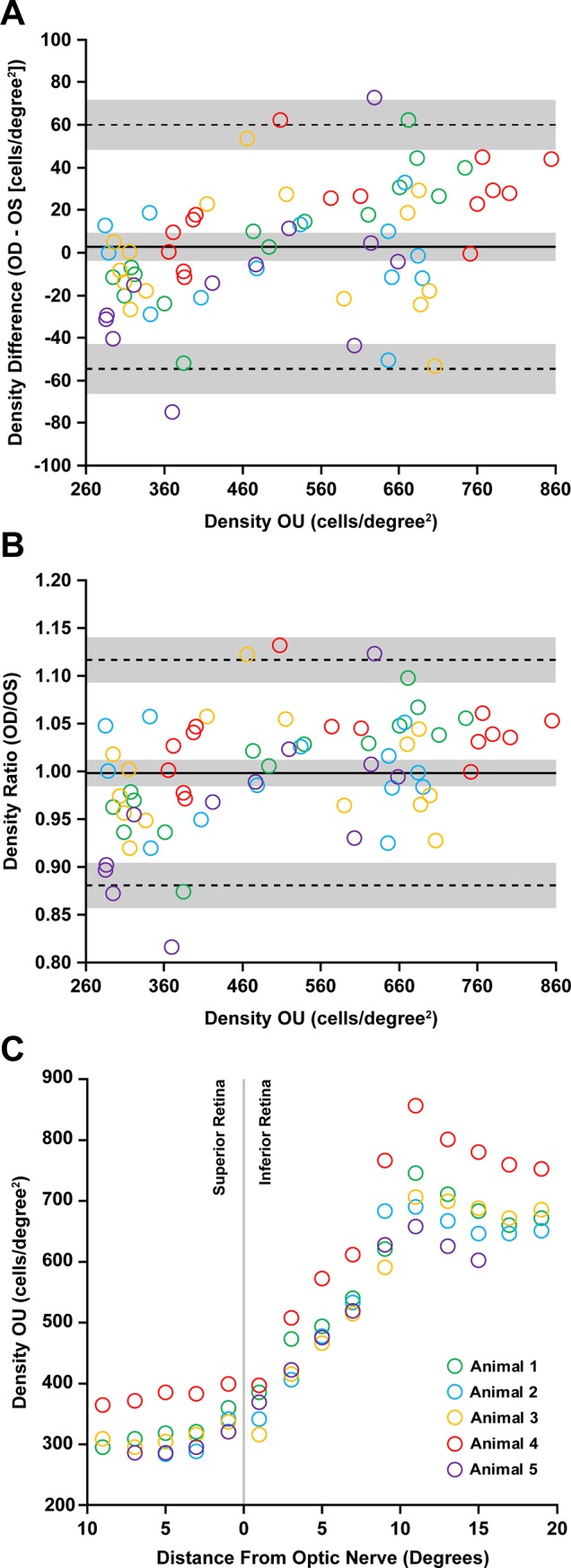
Interocular comparisons of photoreceptor density. (A-B) Bland-Altman plots showing photoreceptor density has interocular symmetry between left (OS) and right (OD) eyes of five 13-LGS (Animals 1–5; legend in (C)) used in this study. Density measurements from all observers were averaged for this comparison. (A) The mean difference between eyes is 2.74 cells/degree^2^ with 95% limits of agreement 60.15 and -54.57 cells/degree^2^. Pearson correlation coefficient (*r*) calculations reveal a significant proportional bias to the magnitude of density (*r* = 0.419, 95% CI = 0.20 to 0.60, *P* = 0.0003). (B) The data were then transformed to highlight the relative interocular symmetry unrelated to mean density differences. The mean ratio of density measurements was 0.99 with 95% limits of agreement 1.12 and 0.88. Thus, the OD and OS values differ by less than 12% for 95% of animals examined. *Solid lines* represent the average difference (A) or ratio (B) between eyes, while *dotted lines* represent 95% limits of agreement. The *gray shading* represents the 95% confidence intervals. (C) Photoreceptor density at distances from the ONH. Left and right eyes were averaged for each data point. Density varies between animals, but the gradient is consistent in these five animals—with low photoreceptor density in the superior retina, and peak density (at the visual streak) 11 degrees inferior to the ONH.

Interocular symmetry of density measurements from AOSLO images of the 13-LGS photoreceptor mosaics was assessed using the average values of all observers. Vertical strip montages (i.e. **Fig 1A**) from both eyes were available for five of eight animals. **Fig 3A** shows the mean differences between eyes, and the bias was close to zero (2.74 cells/degree^2^, 95% CI = 9.602 to -4.122 cells/degree^2^), which is consistent with interocular symmetry. However, as there was a significant relationship between the density difference for the pairs of eyes and the average density for those pairs of eyes (r = 0.42, p<0.0003, Pearson correlation), we plotted the ratio of the right eye (OD) to the left eye (OS) values on the y-axis instead of the OD-OS difference (**Fig 3B**). This is effectively the same as doing a log transformation of the individual values and subtracting them [36]. In this case, the 95% limits of agreement are 0.88 to 1.12 (*dashed lines*, **Fig 3B**). This means that we would expect the OD and OS values to differ by less than 12% for 95% of animals examined. **Fig 3C** shows a consistent density gradient (in degrees^2^) relative to the optic nerve across these five animals. Similar to a previous study [4], the lowest photoreceptors densities (270–409 cells/degree^2^) were found superior to the ONH, and peak photoreceptor densities in the 13-LGS visual streak (656–881 cells/degree^2^) are found 11 degrees (~1.1mm) inferior from the ONH. Thus, while the range of densities between animals varies, the gradient of densities superior and inferior from the ONH are consistent across animals (**Fig 3**). There was not a significant effect of sex on photoreceptor density at any of the 15 regions analyzed (*p* > 0.05, 2-way ANOVA, Sidak’s multiple comparisons test, *n* = 3 males, 5 females). There was a significant effect of age on photoreceptor density only in the comparison of 4-month old 13-LGS compared to 15-month old 13-LGS, and only around the visual streak (9 degrees inferior, *p* = 0.021; 11 degrees inferior, *p* = 0.001; 13 degrees inferior, *p* = 0.020; 2-way ANOVA, Sidak’s multiple comparisons test, *n =* 3 at the age of 4 months, 2 at the age of 15 months, 3 at the age of 27 months).

## Discussion

The ability to reliably assess the cone-dominant photoreceptor mosaic of the 13-LGS *in vivo* is important as models of cone disorder are developed in this species. Confocal AOSLO is limited in the detection of single 13-LGS photoreceptors because of their variable multimodal reflective appearance [4], which is also seen in perifoveal human cones [38]). However, split-detection AOSLO can more clearly resolve all photoreceptors imaged in the 13-LGS compared to confocal AOSLO [4]), and limited eye movement of anesthetized animals helps register high quality images for analysis (**S1 Video**). Automated detection of photoreceptors (via the adaptive filtering and local detection algorithm [17]) works well on 13-LGS split-detection images of high quality (**Fig 2**). However, manual correction by observers is still needed to add missed photoreceptors or remove erroneously selected photoreceptors. Defining repeatability, reliability, and interocular symmetry of 13-LGS photoreceptor density measurements is critical to effectively assess any photoreceptor phenotypes in future transgenic models in this species, or experimental models that use one eye as a control.

As expected, interocular symmetry was observed with photoreceptor density measurements, with mean difference between eyes being approximately zero (**Fig 3A**) and the ratio of both eyes being approximately 1:1 (**Fig 3B**). The Bland-Altman analysis indicates that a density difference between eyes of 12% or more would be considered significantly different. The magnitude of density measurements showed proportional bias (**Fig 3A**), which highlights a methodological limitation that interocular measurements were not collected at the same position along the horizontal meridian. These results suggest that cell density may not be as uniform across the ground squirrel visual streak as we assumed in this study (based on California ground squirrel topography maps [39, 40]). Precise topographical distribution of photoreceptors is unknown in the 13-LGS. Interocular symmetry of axial distance measurements between hyper-reflective bands that correspond to the photoreceptors and RPE in this species was seen with OCT [24]), suggesting that an experimental paradigm involving a contralateral control eye to assess photoreceptor changes in the 13-LGS is a valid approach. Photoreceptor density in this study ranged from 166–881 cells/degree^2^, which corresponds to 16,562–87,902 cells/mm^2^ if a retinal magnification factor of 100 μm/degree is used (based on the 7.90 mm axial length of the European GS [41])). This density range is similar to the California ground squirrel [39, 40] and our initial 13-LGS AOSLO report [4]). A limitation of the present study is that axial length was not measured in these animals. As axial length is known to affect lateral scale of in vivo retinal images [42, 43], we reported cell density in cells/degree^2^. Human AOSLO images are often scaled linearly by the subject’s axial length using an assumed 291 μm/degree retinal magnification factor derived from the 24 mm Gullstrand model eye [44]. An established model eye does not exist for the 13-LGS but should be derived to reduce some assumptions in scaling of non-invasive retinal images. Once lateral scaling can be more accurately approximated in individual 13-LGS, it will be possible to derive a more complete understanding of the range of photoreceptor densities in 13-LGS as a function of age and sex. For example, while no differences in cone density were detected between sexes in our study, the extent to which axial length and photoreceptor density varies between animals and sexes is unknown. As such, we cannot rule out the possibility of sex-based differences in areal cone density measurements. It is possible that observed effects of age on photoreceptors are due to eye growth rather than a real change in photoreceptor density. In addition, similar comparisons will be needed to examine possible differences between wild-caught and captive-bred animals.

Intraobserver repeatability ranged from 9.43 to 25.71 cells/degree^2^ (1.99–5.55%) in this study (**Table 2**), and these data suggest that the difference between two density measurements from 13-LGS images captured from our AOSLO should be less than 28 cells/degree^2^ for 95% of observations (**Table 2**). While we used images the span the range of typical 13-LGS image quality, increased image quality results from the stability of anesthetized 13-LGS subjects compared to actively fixating human volunteers with varying levels of fixational stability. However, this study used only non-confocal split-detection AOSLO images, whereas many of the repeatability studies of human cone density used confocal AOSLO in which the appearance of individual cones can be more variable [5, 6, 11]. Cone density measurements using confocal AOSLO images captured from a population of healthy individuals using a similar semi-automated method were similar in intraobserver repeatability (2.7%) [5] and interobserver reliability (ICC of 0.957) [6] to this study.

Our assessment of interobserver reliability suggests that not all observers perform alike. Observers 3 and 5 were significantly different in their measurements than the observers 1, 2, and 4, but not different from each other. Since observers 3 and 5 had repeatable measurements (**Table 2**), this result suggests that these observers corrected cell identification outputs differently than the other 3 observers. Previous work has showed poor interobserver reliability related to experience in cell identification [9]. Additionally, observer experience was not considered in the data analysis but may be a source of variance in these results. For example, observer 1 was the most experienced 13-LGS photoreceptor identifier, whereas observer 2 was the least experienced in identifying 13-LGS photoreceptors. The number of years in an imaging lab performing these tasks may be related to the repeatability and reliability performance in this study; Observer 1 was the most senior (5 years), followed by observer 5 (3 years), observer 2 (2 years), observer 3 (1 year), and observer 4 (<1 year). This level of experience is nearly identical to the performance in repeatability (**Table 2**). Our results highlight the importance of observer training and accurate automation for AOSLO cell identification.

Interobserver agreement between cone density measurements was worse in lower quality images (**Fig 2**). When quantitative assessments of image quality were performed in this study, the images one might qualitatively determine as high or low quality often matched the scores in the CellProfiler metrics (**S2 Fig**). Since this method scores images based on blur and pixel intensity metrics, it may not be appropriate for all images of damaged or disease photoreceptors, where the images can have high contrast but photoreceptor mosaic structure is changed or unrecognizable. This is particularly relevant to 13-LGS retinal imaging were some photoreceptor images from torpid animals have high contrast and low blur but are unrecognizable as photoreceptors [45]. Therefore, when counting photoreceptors, computational methods involving training datasets and machine learning should be developed for the appropriate animal model or retinal disorder to accurately automate cell identification. This approach has been recently applied to finding cones in subjects with achromatopsia [20] and Stargardt disease [21]. Image quality variability with *in vivo* imaging is common if the tear film of the subject is not meticulously maintained for wavefront correction using adaptive optics. Methods to reject blurry image collection may be warranted to increase repeatability of density and other photoreceptor mosaic metrics. In addition, future studies may determine a threshold image quality above which reliable cone density estimates can be obtained, which could significantly enhance longitudinal studies in these animals.

In conclusion, we have characterized the interocular symmetry (**Fig 3**), intraobserver repeatability (**Table 2**, **S1 Fig**), and interobserver reliability of 13-LGS photoreceptor density measurements from images collected with our AOSLO system using the adaptive filtering and local detection algorithm [17]. This algorithm performed well on 13-LGS split-detection AOSLO images and led to excellent repeatability of observers to correct the cell identification output of the algorithm. Our analysis was limited to 30 degrees in the posterior pole, which includes the cone-rich visual streak. True peak cone density could be found elsewhere along the visual streak horizontal meridian, but we have found peak cone density to be consistently located 11 degrees inferior from the ONH (~1.1 mm). While this is a promising start for assessing repeatability and reliability of photoreceptor metrics in animal models, our work is limited to one measurement metric from images collected by one AOSLO system in one animal species. Validation of this method in 13-LGS will come with additional studies using custom or commercial AO systems in additional lab environments working with additional species. Overall, this method could be used to monitor longitudinal changes in photoreceptor density in the 13-LGS, though attention to image quality will be required for reliable data collection.

## Supporting information

S1 Dataset Observer measurements. (XLSX)

S1 Fig Standard deviation of observer measurements. Mean and standard deviations from all 15 measurements from all five observers (top left panel), and mean and standard deviations from the three measurements from each observer (remaining five panels). Despite the range of photoreceptor densities, there was not a significant trend when comparing the total 15-observation (from all observers) mean photoreceptor density and standard deviation for all 214 images (p = 0.456, linear regression).
(PDF)

S2 Fig Distribution of image quality. Results from CellProfiler analysis of image quality from all 214 images, and the images with Min, Median, and Max score for each metric. (A) ‘Focus Score’ measures pixel intensity variance across the image using a normalized variance algorithm. (B) ‘Power Log-Log Slope’ measures the slope of the image log-log power spectrum. (C) ‘Std Intensity’ measures the standard deviation of pixel intensity values. (D) ‘MAD Intensity’ measures the median absolute deviation of pixel intensity values.
(PDF)

S1 Video Raw and registered video of 13-LGS photoreceptors. (AVI)
