# Supplementary material for: Interocular symmetry, intraobserver repeatability, and interobserver reliability of cone density measurements in the 13-lined ground squirrel
Source: PLoS One. 2019 Sep 26;14(9):e0223110. doi: 10.1371/journal.pone.0223110 (PMC6762077; doi:10.1371/journal.pone.0223110)
Supplement: S1 Fig — Mean and standard deviations from all 15 measurements from all five observers (top left panel), and mean and standard deviations from the three measurements from each observer (remaining five panels). Despite the range of photoreceptor densities, there was not a significant trend when comparing the total 15-observation (from all observers) mean photoreceptor density and standard deviation for all 214 images (p = 0.456, linear regression). (PDF) [file pone.0223110.s002.pdf]

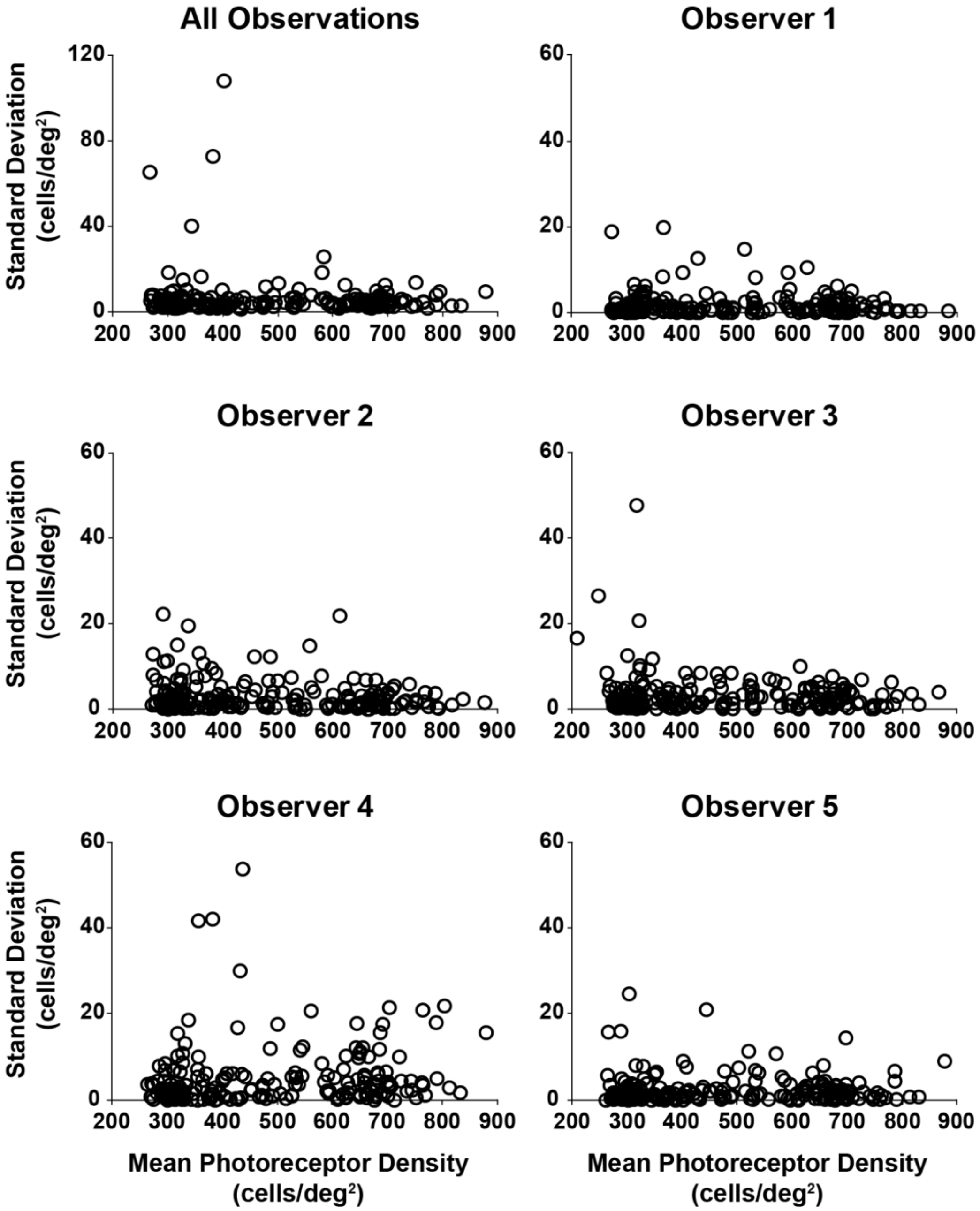

S1 Fig. Standard deviation of observer measurements.

Mean and standard deviations from all 15 measurements from all five observers (top left panel), and mean and standard deviations from the three measurements from each observer (remaining five panels). Despite the range of photoreceptor densities, there was not a significant trend when comparing the total 15-observation (from all observers) mean photoreceptor density and standard deviation for all 214 images ($p = 0.456$, linear regression).
