# Supplementary material for: Interocular symmetry, intraobserver repeatability, and interobserver reliability of cone density measurements in the 13-lined ground squirrel
Source: PLoS One. 2019 Sep 26;14(9):e0223110. doi: 10.1371/journal.pone.0223110 (PMC6762077; doi:10.1371/journal.pone.0223110)
Supplement: S2 Fig — Results from CellProfiler analysis of image quality from all 214 images, and the images with Min, Median, and Max score for each metric. (A) ‘Focus Score’ measures pixel intensity variance across the image using a normalized variance algorithm. (B) ‘Power Log-Log Slope’ measures the slope of the image log-log power spectrum. (C) ‘Std Intensity’ measures the standard deviation of pixel intensity values. (D) ‘MAD Intensity’ measures the median absolute deviation of pixel intensity values. (PDF) [file pone.0223110.s003.pdf]

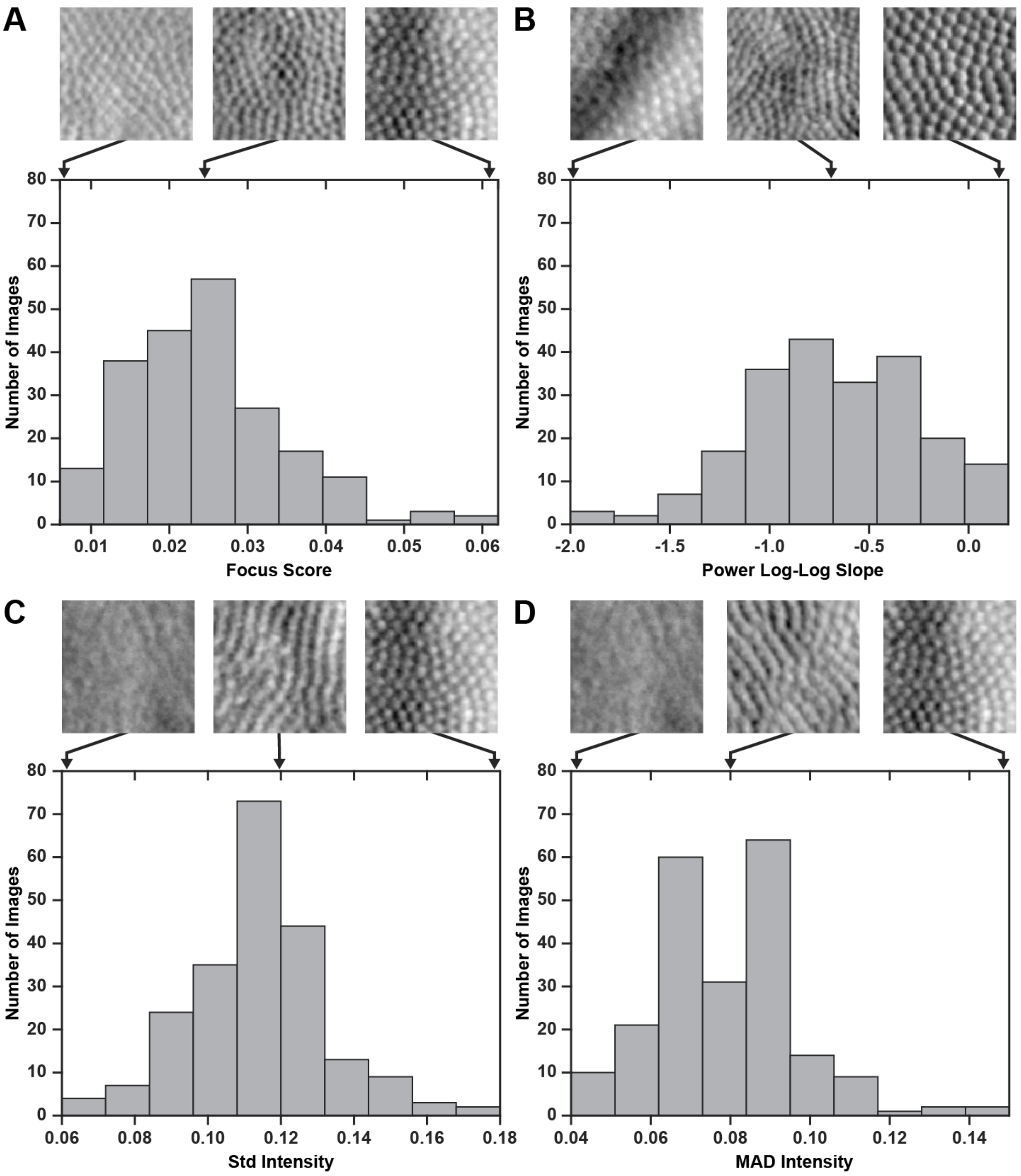

S2 Fig. Distribution of image quality.

Results from CellProfiler analysis of image quality from all 214 images, and the images with min, median, and max score for each metric. (A) 'Focus Score' measures pixel intensity variance across the image using a normalized variance algorithm. (B) 'Power Log-Log Slope' measures the slope of the image log-log power spectrum. (C) 'Std Intensity' measures the standard deviation of pixel intensity values. (D) 'MAD Intensity' measures the median absolute deviation of pixel intensity values.
